# Recommended Immunological Strategies to Screen for Botulinum Neurotoxin-Containing Samples

**DOI:** 10.3390/toxins7124860

**Published:** 2015-11-26

**Authors:** Stéphanie Simon, Uwe Fiebig, Yvonne Liu, Rob Tierney, Julie Dano, Sylvia Worbs, Tanja Endermann, Marie-Claire Nevers, Hervé Volland, Dorothea Sesardic, Martin B. Dorner

**Affiliations:** 1 CEA Saclay, Institute of Biology and Technologies of Saclay, Laboratory for Immunoanalytical Researches, Gif-sur-Yvette 91191 cedex, France; Stephanie.SIMON@cea.fr (S.S.); Julie.Dano@cea.fr (J.D.); Marie-Claire.NEVERS@cea.fr (M.-C.N.); Herve.VOLLAND@cea.fr (H.V.); 2 Biological Toxins, Centre for Biological Threats and Special Pathogens, Robert Koch Institute, Seestrasse 10, 13353 Berlin, Germany; FiebigU@rki.de (U.F.); WorbsS@rki.de (S.W.); tanja.endermann@ayoxxa.com (T.E.); 3 Division of Bacteriology, National Institute for Biological Standards and Control, a Centre of Medicines & Healthcare Products Regulatory Agency, Blanche Lane, South Mimms, Potters Bar, Hertfordshire EN6 3QG, UK; Yvonne.Liu@nibsc.org (Y.L.); Rob.Tierney@nibsc.org (R.T.); Thea.Sesardic@nibsc.org (D.S.)

**Keywords:** proficiency test, botulinum neurotoxin, *Clostridium botulinum*, immunological detection, ELISA, lateral flow immunoassay, endopeptidase, antibodies, matrices

## Abstract

Botulinum neurotoxins (BoNTs) cause the life-threatening neurological illness botulism in humans and animals and are divided into seven serotypes (BoNT/A–G), of which serotypes A, B, E, and F cause the disease in humans. BoNTs are classified as “category A” bioterrorism threat agents and are relevant in the context of the Biological Weapons Convention. An international proficiency test (PT) was conducted to evaluate detection, quantification and discrimination capabilities of 23 expert laboratories from the health, food and security areas. Here we describe three immunological strategies that proved to be successful for the detection and quantification of BoNT/A, B, and E considering the restricted sample volume (1 mL) distributed. To analyze the samples qualitatively and quantitatively, the first strategy was based on sensitive immunoenzymatic and immunochromatographic assays for fast qualitative and quantitative analyses. In the second approach, a bead-based suspension array was used for screening followed by conventional ELISA for quantification. In the third approach, an ELISA plate format assay was used for serotype specific immunodetection of BoNT-cleaved substrates, detecting the activity of the light chain, rather than the toxin protein. The results provide guidance for further steps in quality assurance and highlight problems to address in the future.

## 1. Introduction

Botulinum neurotoxins (BoNTs) are produced by the anaerobic spore-forming bacteria *Clostridium (C.) botulinum* and some strains of *C. butyricum* and *C. baratii*. The bacteria are ubiquitous in the environment and can germinate under suitable conditions to yield the vegetative bacterium that synthesizes the toxin. Among the seven confirmed serotypes (BoNT/A to BoNT/G), serotypes A, B, E, and F are pathogenic to humans [1]. Human botulism is a rare but life-threatening disease that often requires intensive care treatment with extended recovery periods over several months. It is mainly caused by ingestion of food contaminated with botulinum toxin (foodborne botulism), by contamination of a wound with *C. botulinum* spores (wound botulism) or by intestinal colonization and toxin production in infants <1 year old (infant botulism) [2]. Clostridia release their neurotoxins in the form of large protein complexes in culture or food. These complexes consist of the BoNT holotoxin bound to the so-called non-toxic non-hemagglutinin (NTNHA) and, depending on the genetic background, different hemagglutinins [3,4]. These non-toxic accessory proteins shield the BoNT through the harsh gastrointestinal passage and promote the uptake across the intestinal membrane [5,6,7]. Finally, BoNT is taken up by synaptic vesicles at the motor neuronal endplates by dual receptor mediated endocytosis. In the acidified endocytic vesicle the 50 kDa light chain is translocated by the 100 kDa heavy chain into the cytoplasm [8]. Inside the cytosol the light chain, a zinc-dependent endopeptidase, cleaves certain SNARE (soluble *N*-ethylmaleimide-sensitive factor attachment protein receptor) proteins associated with neuronal transmission. While BoNT/A, C, and E target SNAP-25 (synaptosomal-associated protein of 25 kDa), BoNT/B, D, F, and G confer their proteolytic activity on synaptobrevin/VAMP (vesicle-associated membrane protein), each at a distinct site. Only BoNT/C is known to cleave a second SNARE protein, syntaxin [9].

Because of their extreme toxicity and ease of production, BoNTs have been classified by the Centers for Diseases Control and Prevention (CDC) as category A agents [10]. Indeed, the estimated human parenteral and oral lethal doses are 0.1–1 ng/kg and 1 μg/kg, respectively [10], posing a challenge for the confirmation of presence of the toxins in suspected samples [11]. Unlike for infectious diseases, polymerase chain reaction (PCR)-based methods are not considered reliable for BoNT detection, as the organism may not necessarily be present. This can be the case in contaminated beverages or food where the *Clostridia* are heterogeneously distributed or in matrices intoxicated solely with the toxin, accidently or deliberately. Due to its exquisite sensitivity the *in vivo* mouse bioassay is still considered as a gold standard, but it is ethically questionable [12] and time-consuming, providing results within days when rapid diagnosis for implementation of immediate supportive therapy is essential. Animal welfare considerations and the desire for more rapid assays have stimulated renewed efforts to generate or revive specific and sensitive *in vitro* or *ex vivo* detection assays (for review, see [11,12,13]). For example, hemidiaphragm assays have been re-evaluated and are as sensitive as, and considerably faster than, the mouse bioassay, even if they still rely on animal use [14,15]. Neuronal cell based assays present the advantage of being reliable alternative *in vitro* tests, however their sensitivity and applicability to complex matrices might be restricted [16]. Mass spectrometry based assays are very powerful and specific, often combining an immuno-enrichment step to increase sensitivity and to clean the proteins from complex matrices with tryptic digest for protein identification or an endopeptidase assay to assess functional activity [17,18,19,20,21,22,23,24,25]. Overall, all these techniques can reach or exceed the sensitivity obtained with the mouse bioassay but require either complex specialized equipment and/or dedicated technical skills often not available in routine microbiology laboratories.

Antibody-based immunoassays are probably the most commonly used assays performed for BoNT detection. Their ease of use, good specificity (especially when using monoclonal antibodies), high sensitivity, high-throughput capabilities and high speed are some of the reasons for their successful applications in routine laboratories. Different formats have been developed: e.g. enzyme-linked immuno-sorbent assays (ELISA), electro-chemiluminescence-based assays, immuno-PCR, or immuno-chromatographic assays (for review see [11]). ELISA-plate based endopeptidase assays are a relatively new generation of rapid toxin detection methods that combine ease of use with serotype specificity measuring the activity of the toxin rather than its protein concentration [12,13,26,27,28,29,30]. These methods are particularly suited for detection of botulinum toxins formulated for therapy, where traditional immunoassays failed to correlate with the biological activity [31]. Combining detection of endopeptidase activity with the capture of the toxin heavy chain domain also makes this biochemical approach highly suited for detection of toxin in complex matrices such as human serum [27,28,32].

One obstacle in comparing different detection strategies or technical approaches, as well as judging the suitability of a given method, is the lack of standardized reference materials and proficiency tests (PT). To address the latter point and pave the way for the generation of materials that could be developed into a reference material, a PT was performed within the framework of the EU-project EQuATox [33]. A detailed characterization of the BoNT material generated and used in this PT is given by Weisemann *et al.* [34], while an overview of PT results is given by Worbs *et al.* [35], both in this issue of *Toxins* (MDPI, Basel, Switzerland).

In this article, we describe three successful immunological strategies deployed to detect BoNT/A, B and E by different laboratories during the 2013 EQuATox BoNT proficiency test. The EQuATox 2013 international BoNT PT panel consisted of thirteen blinded liquid samples (1 mL each) spiked with BoNT/A, B or E into buffer (0.1% BSA in PBS), cow’s milk, meat (minced pork and beef) extract or human serum at various concentrations, as described in detail by Worbs *et al.* [35]. These three immunological approaches were used to detect, differentiate and quantify the different serotypes in the proficiency panel samples and are presented here as examples of recommended strategies that could be further developed into recommended operating procedures based on their performance in the PT.

## 2. Results and Discussion

### 2.1. Assay Development and Validation

**Figure 1:**
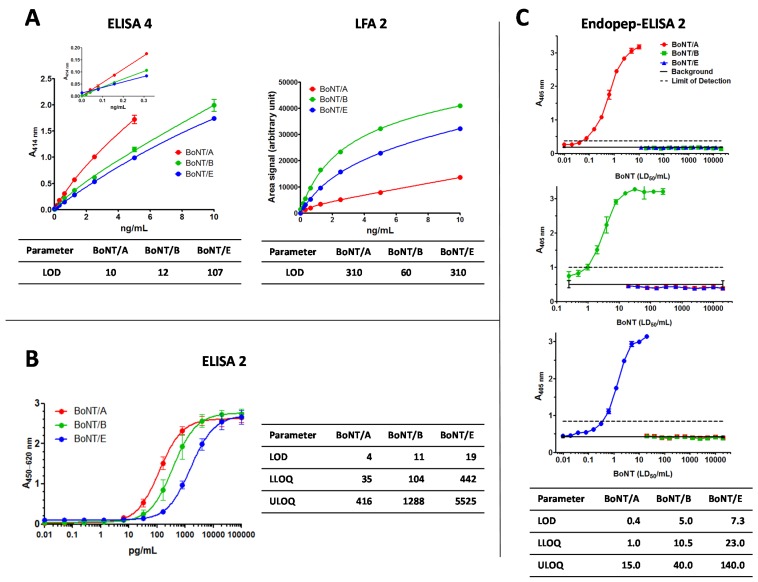
In-house immunoassays used in the botulinum neurotoxin-proficiency test. Validation data for the detection of botulinum neurotoxin (BoNT)/A (red), BoNT/B (green) or BoNT/E (blue) by different assay formats. (**A**) *Commissariat à l’Énergie Atomique et aux énergies alternatives* employed three plate-bound sandwich enzyme-linked immunosorbent assays (ELISA 4) and three lateral flow assays (LFA 2) each specific for the denoted antigen; (**B**) Robert Koch Institute used three different plate-bound sandwich ELISAs (ELISA 2) for the detection of the three BoNTs; (**C**) The National Institute for Biological Standards and Control employed three separate endopeptidase activity assays each specific for one of the three BoNTs (Endopep-ELISA 2). Selected parameters from the validation study are indicated in pg/mL: limit of detection (LOD), lower or upper limit of quantification (LLOQ, ULOQ, respectively). A denotes absorbance at the indicated wavelength.

Laboratories currently rely on different tools, techniques, protocols and reference materials for detection and quantification of BoNTs. Prior to the PT, the participating laboratories developed their own in-house assays adapted to their specific tasks, focusing on the analysis of clinical samples, food, environmental samples or therapeutic preparations. The precise validation of procedures, as well as the scope of validation, depended on different requirements. For the three successful immunological approaches presented in this work, results of the in-house validation studies performed prior to the actual PT are briefly summarized in Figure 1. Please note that the numbering of ELISA, lateral flow assay (LFA), and Endopep-ELISA formats follows the numbering in Worbs *et al.* [35].

#### 2.1.1. ELISA 4 and LFA 2

The approach “ELISA 4” was developed by the *Commissariat à l’Énergie Atomique et aux énergies alternatives* (CEA), Centre de Saclay, France: herein, monoclonal antibodies directed against the *C*-terminal fragment of the BoNT heavy chain were used in the three independent ELISA to detect BoNT/A, B or E, respectively. Theoretical limits of detection (LOD) and of quantification (LOQ) have been calculated by means of eight negative control signals plus three standard deviations (SD) or plus 10 SD, respectively, and are 10 pg/mL and 30 pg/mL for BoNT/A, 12 and 50 pg/mL for BoNT/B, and 107 and 500 pg/mL for BoNT/E, using commercially available BoNT standards (purified holotoxins) from Metabiologics Inc. (Madison, WI, USA). In the quantitative working range of the ELISA the coefficients of variation (CV%) are lower than 25% and the recoveries lie between 70% and 112%. Intra-assay and inter-assay CV% were inferior to 8% and 11%, respectively, at a concentration of 1 ng/mL of toxin, with *n* = 5 as the number of intra- or inter-assay repetitions performed in six replicates. Cross-reactivity (calculated as the percentage of BoNT of interest concentration/other BoNT concentration detected for a given absorbance) was less than 0.005%. All three ELISAs were able to detect purified holotoxins as well as BoNT-complexes.

LFA 2 developed by CEA: Monoclonal antibodies used in the three assays for BoNT/A, B and E are also directed against the *C*-terminal fragment of the BoNT heavy chain. They were not necessarily the same as the ones used in the sandwich ELISA. They have been selected specifically for their sensitivity in the LFA format, all against both the corresponding holotoxin and BoNT-complex. LODs have been calculated using commercially available standards (purified holotoxins) from Metabiologics and an ESE Quant reader, and are 310 pg/mL, 60 pg/mL, and 310 pg/mL for BoNT/A, B, and E, respectively. No cross-reactivity was detected. Modified versions of these tests are available commercially from NBC-sys (Saint-Chamond, France), with LOD of 5 ng/mL for each of the three toxins.

Standard curves for the three ELISAs and LFAs are shown in Figure 1A with data for BoNT/A given in red, for BoNT/B in green, and for BoNT/E in blue.

#### 2.1.2. ELISA 2

At Robert Koch Institute (RKI), Germany, a panel of monoclonal and polyclonal antibodies against different BoNTs have been developed and implemented into different technical applications [36,37,38,39,40,41]. For screening of food matrices a multiplex magnetic bead-based suspension array based on the Luminex xMAP platform (Austin, TX, USA) was established which simultaneously detects BoNT/A, B, ricin, abrin, and staphylococcal enterotoxin B (SEB) [37]. Prior to the PT, the suspension array was extended into a six-plex assay format to cover all serotypes pathogenic to humans (BoNT/A, B, E, and F), plus ricin and SEB. The latter two served as internal assay controls for undesired matrix interferences when suspected botulism samples were analyzed (e.g. background signals and aggregation of beads).

For precise quantitation, conventional plate-bound sandwich singleplex ELISAs were established and validated. Absorbance was plotted against the logarithmic concentration and fitted against a sigmoidal dose response curve. Individual ELISAs were performed three to five times in duplicate and standard curves for the three ELISAs are shown in Figure 1B. The ELISA for BoNT/A is depicted in red, for BoNT/B in green, and for BoNT/E in blue (Figure 1B). The LOD as well as the lower and upper limits of quantification (LLOQ, ULOQ) of the three sandwich ELISAs were determined from the regression curves and are also given in Figure 1B. ELISA for BoNT/A was found to be the most sensitive with an LOD of 4 pg/mL and an LLOQ of 35 pg/mL followed by the BoNT/B ELISA (LOD: 11 pg/mL; LLOQ: 104 pg/mL). The least sensitive was the one for BoNT/E with an LOD of 19 pg/mL and an LLOQ of 442 pg/mL. All ELISA formats detect BoNT holotoxin as well as BoNT in its different complexes. There was no observed cross-reactivity with other BoNT serotypes at concentrations around the effective half-maximal concentration (EC_50_). Matrix compatibility was successfully tested for beverages, food matrices and human serum. The combined approach of the three individual ELISAs for BoNT/A, B and E which was used in the PT is entitled “ELISA 2”.

#### 2.1.3. Endopeptidase-ELISA 2

Over the past fifteen years The National Institute for Biological Standards and Control (NIBSC), United Kingdom, has developed a range of endopeptidase assays specific for BoNT/A, B, C, and E by generating anti-peptide antibodies targeting the newly exposed epitopes on either SNAP-25 or VAMP2 peptides [26,42,43] generated after cleavage by toxins. As each of these neurotoxins cleaves a specific bond within their target protein, such assays are entirely serotype specific. Sensitivity is often superior to the mouse bioassay with detection of 0.08 median lethal dose (LD_50_)/mL (~0.4 pg/mL) and 0.3 LD_50_/mL (~7 pg/mL) reported for BoNT/A and BoNT/E, respectively. Whereas sensitivity of the assay can be further increased for BoNT/A to 0.01 LD_50_/mL (~40 fg/mL) in the presence of 0.1% albumin [43], higher concentrations of albumin are known to interfere in these assays due to proteolysis of SNAP-25 [44]. Endopeptidase assay for BoNT/B, with sensitivity of 1 LD_50_/mL (~5 pg/mL) was of the lowest sensitivity, but it is still as sensitive as the mouse bioassay.

Both purified holotoxins as well as their complexed forms could be detected equally with endopeptidase assays. Typical standard curves with the three reference toxins and serotype specificity for the three endopeptidase assays are shown in Figure 1C, using LD_50_ activity for dose response. Limit of detection (LOD), and upper and lower limit of quantification (LLOQ and ULOQ) were calculated from the regression curves and are also given in Figure 1C, which were expressed in ng/mL, calculated from the provided specific activity for each toxin (information on specific activity in LD50/mg can be found in the experimental Section 3.3.2). Inter-assay and inter- and intra-plate variability was calculated and determined to be less than 5%. Matrix effect, other than of human serum albumin in BoNT/A assay, was not previously studied. BoNT/A endopeptidase assay has been validated at NIBSC for particular brands of therapeutic BoNT/A for injection [42], where the activity per vial is determined relative to a product specific reference, or an in house reference, calibrated in mouse LD_50_ [45]. This PT study provided for the first time an opportunity to use these endopeptidase assays in the field of toxin detection.

### 2.2. The Proficiency Test Scheme

The composition of the 13 PT samples is presented in Table 1. The samples contained various concentrations of BoNT/A, B or E in buffer (0.1% BSA in PBS), meat extract, cow’s milk or human serum in a 1 mL volume. Matrices were chosen on the basis of an *a priori* homogeneity and stability testing [35]. Occasional intoxications can occur by strains producing more than one serotype [9]. To mimic intoxication by a bivalent strain, sample S8 contained two serotypes (BoNT/A plus B) in similar amounts. The nominal concentration for each sample has been determined by the organizing laboratory using ELISA and recombinant BoNT standard materials produced for the EQuATox project [34].

**Table 1:** Composition of proficiency test samples.

Sample	Matrix	Serotype	*x*_a_ Nominal Concentration (ng/mL)
**S1**	meat extract	BoNT/A	10.5
**S2**	0.1% BSA/PBS	BoNT/A	9.9
**S3**	0.1% BSA/PBS	none	0.0
**S4**	0.1% BSA/PBS	BoNT/E	10.9
**S5**	meat extract	BoNT/A	108.0
**S6**	0.1% BSA/PBS	BoNT/B	9.0
**S7**	0.1% BSA/PBS	BoNT/A	100.0
**S8**	0.1% BSA/PBS	BoNT/A	4.7
		BoNT/B	4.5
**S9**	0.1% BSA/PBS	BoNT/A	0.5
**S10**	cow’s milk	BoNT/A	10.3
**S11**	human serum	BoNT/A	9.8
**S12**	0.1% BSA/PBS	BoNT/A	1001.0
**S13**	cow’s milk	BoNT/A	112.0

### 2.3. Overview of the Diagnostic Approaches Utilized by the Participating Laboratories

**Figure 2:**
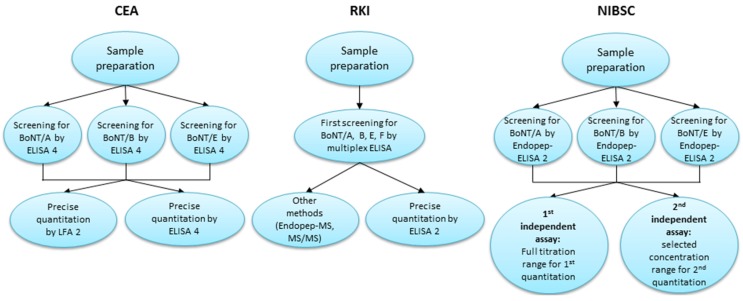
Diagnostic approaches for proficiency test sample analysis by three different laboratories. After sample preparation *Commissariat à l’Énergie Atomique et aux énergies alternatives* (CEA) used three selective enzyme-linked immunosorbent assays (ELISAs) for botulinum neurotoxin (BoNT)/A, B and E (ELISA 4) for first screening in order to detect serotypes present in each sample. For the serotypes detected during screening, precise quantitation was achieved on selected samples by the corresponding ELISA 4 and lateral flow assay (LFA) 2. Robert Koch Institute (RKI) employed a multiplex ELISA for screening to detect serotypes present and for tentative quantitative assessment. Precise quantitation was performed for the identified serotypes by the corresponding ELISA specific for BoNT/A, B or E (ELISA 2). Using parallel samples, endopeptidase-mass spectrometry (Endopep-MS) assay was performed to corroborate ELISA data and one sample was also subjected to tandem mass spectrometry (MS/MS) for unambiguous identification. The National Institute for Biological Standards and Control (NIBSC) used their serotype-specific endopeptidase-ELISA (Endopep-EISA 2) first for screening of samples and using a full titration range for quantification (first result). For the second independent result only selected dilutions were quantified in another run (second result).

The three participating laboratories involved in this article utilized various approaches and techniques for sample analysis during this study. A schematic overview of these approaches is presented in Figure 2. The challenges were to provide accurate qualitative and/or quantitative results, with correct serotype identification from two independent measurements for each sample with only 1 mL of sample available.

#### 2.3.1. Diagnostic Approach of CEA

For CEA laboratory, the first screening was performed using three individual sandwich ELISA (ELISA 4) for the three serotypes (A, B and E) independently after four-fold and 16-fold dilutions of all samples (use of 210 μL of each sample). Purified holotoxins from Metabiologics were used as standards to determine concentrations of the toxins in the samples. Each sample found negative for one or more toxin was not retested for these toxins, except S3 (found negative for the three serotypes). All samples found positive for one or two toxins were retested at least once by sandwich ELISA for the positive toxins at different dilutions (from four-fold for S4, S8 and S9 to 400-fold for S12), in order to be in the linear working range of the standard curve. Based upon the results obtained in the first screening with sandwich ELISA, all the samples were tested using one of the three specific LFA (LFA 2), from at least a two-fold dilution (60 μL of sample) to an 80-fold dilution. After visual observation confirming a qualitative result (presence or absence of the toxins), strips were read with an ESE Quant reader and quantification was performed using a standard curve (Figure 1A). In all cases except S3 (negative in the three tests), samples were retested. In the case of S8, containing a mixture of BoNT/A and B, the sample was tested twice in each test, at a two-fold dilution. Overall the total volume used to perform all the tests (sandwich ELISA and LFA) for one sample was up to 770 μL.

#### 2.3.2. Diagnostic Approach of RKI

In light of the restricted sample volume of 1 mL, RKI employed an in-house developed immunological six-plex magnetic bead-based suspension array based on the Luminex xMAP technology to test for the presence of the BoNT serotypes pathogenic to humans (BoNT/A, B, E, and F). This assay was used not only as a screening tool to achieve a qualitative assessment of the serotypes present in each of the samples, but also to estimate the quantity of analytes. Samples (50 μL) were tested undiluted followed by four serial 10-fold dilutions (1:10 to 1:10,000) using 60 μL of sample volume in total. Based on the results from the initial screening, samples were subjected to three conventional plate-bound singleplex sandwich ELISAs for precise quantification. The combined ELISA approach of the three individual ELISA against BoNT/A, B, and E is named “ELISA 2” and was used for the reporting of qualitative and quantitative results within this PT. In parallel, samples were analyzed by different mass spectrometric approaches (tryptic digest followed by identification of peptide fingerprint; Endopep-MS approach) as depicted in Figure 2.

Depending on the first estimation of concentration, samples were tested in six consecutive two-fold dilutions using up to 62.5 μL sample volume in the corresponding conventional singleplex sandwich ELISA (ELISA 2). Sample S8, which was found positive for BoNT/A and B, was tested by two independent ELISAs. Analyte concentrations were calculated based on dilutions that lay in the linear range (between LLOQ and ULOQ) of the sigmoidal dose response curve. Commercially available BoNT (holotoxin) preparations from Metabiologics were used as reference materials. Analysis was repeated to obtain two independent results for reporting using up to 125 μL of sample volume in total. The whole immunological approach utilized less than 200 μL of sample to achieve qualitative and quantitative results.

#### 2.3.3. Diagnostic Approach of NIBSC

Three separate *in vitro* endopeptidase serotype specific immunoassays were used to screen the test samples for the presence of BoNT/A, B or E toxins (Endopep-ELISA 2). For the first independent assay, all samples were diluted 1:1 (using 100 μL of each test sample per assay) in the assay specific reaction buffer and doubling dilutions were performed across the substrate coated 96 well polystyrene ELISA plates to obtain a full titration dose range. For the second independent assay, using the results from the full titration range obtained in the first assay, the positive samples were diluted to an optimal starting dilution, which was between 1:16 and 1:4096, depending on the test sample (Figure 2). All the negative samples were again diluted 1:1 in the assay reaction buffer to confirm the results from the first screening.

Each assay and ELISA plate included purified reference BoNT/A, B or E holotoxin (Figure 1C) from Metabiologics in order to calculate the relative toxin concentration in the test samples by a parallel line analysis, taking the points within the linear range of the log transformed data. All three assays were performed on at least two separate occasions up to 13 days apart using a total of ~600 μL per test sample to complete all the assays and to achieve both qualitative and quantitative results.

### 2.4. Individual Laboratory Assay Results

Based on their in-house assays, each of the three participating laboratories followed their own strategies to analyze the PT samples. The individual results obtained by the different immunoassays are described below.

#### 2.4.1. Laboratory Results from CEA

ELISA 4: The first sandwich ELISA screening allowed us to correctly assign BoNT/A, B, and E qualitatively for 12 out of 13 samples. Sample S12 was assigned correctly for BoNT/A (presence) and E (absence) but not for BoNT/B, for which the sample was reactive and just above the LOD. Sample S3 was negative for the three toxins tested. As BoNT/F has not been tested, its presence could not been ruled out, sample S3 has therefore been assigned as “not analyzed” instead of “absence of BoNT”. Based on the absorbances measured and compared to the standard curves in this first screening, samples were retested (if needed) with appropriate dilutions and measured against purified BoNT holotoxins from Metabiologics as standard. All the measurements were performed to obtain two independent results as specified by the PT reporting protocol.

LFA 2: Dilutions of the samples were performed from the results obtained in the first ELISA screening. Samples were tested twice independently and read by naked eye (giving qualitative results) or by an ESE Quant reader allowing the quantification (area of the signal obtained for the test line compared to those obtained for the standard curve). As for ELISA, sample S3 was assigned as “not analyzed” instead of “absence of BoNT”, because we did not test for the presence of BoNT/F and could not rule out its presence. Except for this, all the samples have been correctly assigned.

#### 2.4.2. Laboratory Results from RKI

For qualitative and tentative quantitative measurements, PT samples were analyzed by an in-house six-plex suspension array using 60 μL of sample. This first screening allowed correct qualification of BoNT/A, B, and E for all 13 samples, while BoNT/F was not detected in any of the PT samples. BoNT/A was found in samples S1, S2, S5, S7, S8, S9, S10, S11, S12, and S13. Whereas BoNT/B was detected in samples S6 and S8, BoNT/E was only found in sample S4. Sample S3 did not return positive results for any of the toxins tested and was therefore assigned as negative PT control sample. Qualitative findings were reported as positive or negative for the presence of BoNT and again individually for each of the four serotypes. Based on the observed estimated concentration, samples were diluted appropriately and measured against the standard, purified BoNT holotoxins from Metabiologics in conventional plate-bound ELISA (ELISA 2), again using 60 μL of sample. The measurement was repeated to obtain two independent results as specified by the PT protocol.

ELISA results were confirmed by an Endopep-MS approach as previously described [38,46] but with in-house generated mAb against BoNT/A, B, and E. For the sample with the highest BoNT/A concentration (S12) it was possible to achieve unambiguous identification of BoNT/A1 by an MS/MS approach (Figure 2).

#### 2.4.3. Laboratory Results from NIBSC

For qualitative and quantitative findings, PT samples were analyzed in three separate in-house endopeptidase assays specific for BoNT/A, B or E toxins (Endopep-ELISA 2). Assay for BoNT/F was not considered sufficiently developed and was not included in this study. The first set of independent assays, in which all samples were analyzed after 1:1 dilution, using 100 μL of each sample for the test, provided information on qualitative findings for toxin positive and negative samples. Ten of 13 test samples tested positive for BoNT/A toxin, two (S6 and S8) tested positive for BoNT/B, with S11 indicating background interference. Two samples (S4 and S11) also tested positive for BoNT/E, although S11 contained only BoNT/A in human serum. Sample S3 returned negative signals in all three assays, but this sample did not contain undiluted human serum so it could not serve as a negative control for sample S11. For quantitative findings a second assay was performed on PT samples diluted from the tentative concentration calculated in the first assay and measured against individual purified BoNT holotoxin. The measurements were repeated to obtain two valid independent results, as specified by the PT protocol. When reporting quantitative results for BoNT/A, a statistically significant (*p* = 0.0003, *n* = 10) lower value was measured in the second assay, which correlated with the length of time between the two testing (range 6 to 13 days), suggesting a potential instability of toxin on prolonged storage.

### 2.5. Overall Assessment of Immunological Results

#### 2.5.1. Qualitative Results

From the history of botulism outbreaks it is clear that most outbreaks are associated with serotypes A or B. Outbreaks caused by BoNT/E are occasionally observed in countries in the northern hemisphere (e.g., Canada, Japan, Russia, and Scandinavia), while outbreaks caused by BoNT/F are extremely rare [47]. Therefore, the majority of developed assays cover the most prevalent toxins, BoNT/A and B [11]. For qualitative reporting, laboratories were expected to report the presence or absence of BoNT (unspecified) and in addition the presence of any of the four serotypes BoNT/A, B, E, and F. Due to the serotype restrictions of the methods used, and the limitation in sample volume, a number of participants did not address BoNT/F, including two of the laboratories reporting here. The PT organizer evaluated the reported data and assigned a color code for the individual qualitative reporting summaries. Table 2 shows the results of the qualitative analyses for three out of 23 total participants. Here, green denotes a correct assignment, which can be either correct positive (“1”) or correct negative (“−1”), whereas false positive (“10”) or false negative (“−10”) results are highlighted in red. Blank cell indicates that either result was not available or sample was not analyzed.

As can be seen in Table 2, the immunological methods employed delivered very good qualitative results with little or no incorrect findings with two notable exceptions: Test sample S12 (1001 ng/mL BoNT/A in 0.1% BSA/PBS) and S11 (9.8 ng/mL BoNT/A in human serum). In ELISA 4 sample S12 with the highest BoNT/A concentration returned a very low, but considered positive signal for BoNT/B. This could be a result of cross-reactivity of one or both antibodies towards the related BoNT/A molecule, which shares 37.5% identity at an amino acid level [48]. However, cross-reactivity experiments, prior to the PT, showed no cross-reactivity with BoNT/A using BoNT/B sandwich ELISA 4 (against 2 μg/mL of BoNT/A from Metabiologics). When sample S11 was analyzed by the Endopep-ELISA 2 negative results but high background interference were observed for BoNT/B and a truly false positive for BoNT/E, which was confirmed post PT study to be due to human serum effect on the SNAP-25 substrate.

**Table 2:** Selected qualitative PT results ^a^.

Method	PT Sample	BoNT	BoNT/A	BoNT/B	BoNT/E	BoNT/F
**LFA 2**	S1	1	1	−1	−1	-
	S2	1	1	−1	−1	-
	S3	^b^	−1	−1	−1	-
	S4	1	−1	−1	1	-
	S5	1	1	−1	−1	-
	S6	1	−1	1	−1	-
	S7	1	1	−1	−1	-
	S8	1	1	1	−1	-
	S9	1	1	−1	−1	-
	S10	1	1	−1	−1	-
	S11	1	1	−1	−1	-
	S12	1	1	−1	−1	-
	S13	1	1	−1	−1	-
**ELISA 4 ^c^**	S1	1	1	−1	−1	-
	S2	1	1	−1	−1	-
	S3	^b^	−1	−1	−1	-
	S4	1	−1	−1	1	-
	S5	1	1	−1	−1	-
	S6	1	−1	1	−1	-
	S7	1	1	−1	−1	-
	S8	1	1	1	−1	-
	S9	1	1	−1	−1	-
	S10	1	1	−1	−1	-
	S11	1	1	−1	−1	-
	S12	1	1	10	−1	-
	S13	1	1	−1	−1	-
**ELISA 2**	S1	1	1	−1	−1	−1
	S2	1	1	−1	−1	−1
	S3	−1	−1	−1	−1	−1
	S4	1	−1	−1	1	−1
	S5	1	1	−1	−1	−1
	S6	1	−1	1	−1	−1
	S7	1	1	−1	−1	−1
	S8	1	1	1	−1	−1
	S9	1	1	−1	−1	−1
	S10	1	1	−1	−1	−1
	S11	1	1	−1	−1	−1
	S12	1	1	−1	−1	−1
	S13	1	1	−1	−1	−1
**Endopep-ELISA 2**	S1	1	1	−1	−1	-
	S2	1	1	−1	−1	-
	S3	−1	−1	−1	−1	-
	S4	1	−1	−1	1	-
	S5	1	1	−1	−1	-
	S6	1	−1	1	−1	-
	S7	1	1	−1	−1	-
	S8	1	1	1	−1	-
	S9	1	1	−1	−1	-
	S10	1	1	−1	−1	-
	S11	1	1	^b^	10	-
	S12	1	1	−1	−1	-
	S13	1	1	−1	−1	-

^a^ qualitative results are denoted as: 1 = true positive, −1 = true negative, 10 = false positive, - = not analyzed/not available; ^b^ results have not been clearly reported as negative according to the PT scheme and were therefore not displayed here; ^c^ results of ELISA 4 have been deduced from the laboratory’s quantitative reporting, since they accidentally have not been reported qualitatively.

Overall, the qualitative detection by immunoassays was quite robust regardless of the individual technology used (ELISA, LFA, or Endopep-ELISA), not only for the three individual participating laboratories, but throughout all results obtained by immunological methods (Please refer to Worbs *et al.* [35]).

#### 2.5.2. Quantitative Results

For quantitative reporting, participants were asked to submit concentrations in ng/mL from two independent measurements. The individual PT reports sent out to every participant stated the nominal concentration (assigned value, *x*_a_), the reported concentration (*x*) and the *z*-score. In particular, the *z*-score allows for comparison of the closeness of the results with the assigned value independent of analyte, test material or analytical method. The *z*-score indicate by how many standard deviations an observed value deviates from the assigned value transformed to a standard normal distribution [49]. For more information please refer to Worbs *et al* [35] in this issue of *Toxins*. Table 3 lists the assigned values (*x*_a_), the observed values (*x*) and *z*-scores of the quantitative results obtained by the three participating laboratories for BoNT/A, B, and E, in this study.

**Figure 3:**
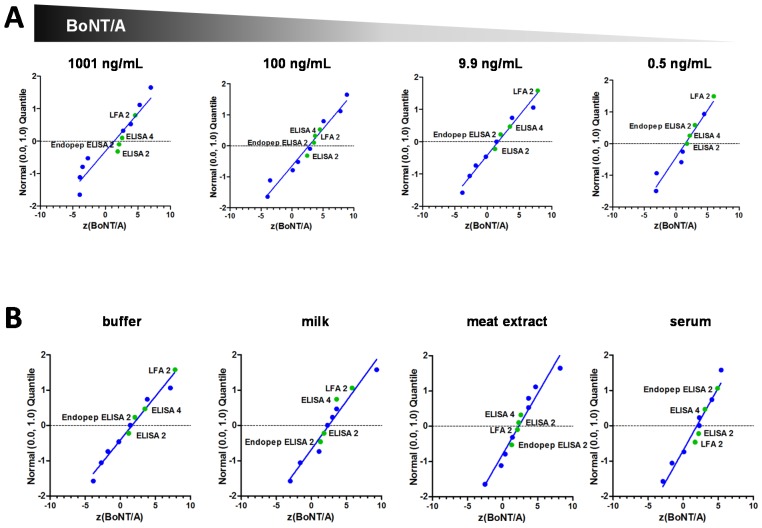
Normal probability plots of *z*-scores for selected results. *z*-scores obtained by various methods and different participants are shown in blue while *z*-scores of the three reporting laboratories are shown in green. Blue line represents linear regression analysis of all *z*-scores. (**A**) *z*-scores achieved with decreasing concentrations (1001, 100, 9.9, and 0.5 ng/mL) of BoNT/A in buffer. (**B**) Laboratories *z*-scores for approximately 10 ng/mL BoNT/A in buffer, milk, meat extract or human serum.

The litmus test for any analytical method is how close the observed value is with the assigned value. Here the *z*-score provides valuable information on the analytical quality achieved by the participating laboratory and Table 3 lists the corresponding *z*-scores for the measured concentrations. If the same analytical method and reference material is used by all participants the observed values should fit a Gaussian distribution, and this means that a *z*-score between −2 and 2 would be expected for 95% of the observed values. In analytical chemistry *z*-scores between −2 and 2 are usually designated “satisfactory”, whereas a *z*-score greater than 3 or below −3 would be expected for only 0.1% of the (standard normal distributed) results and is therefore attributed “requiring action” [49]. Such stringent criteria, which are often applied in PTs performed in analytical chemistry on small chemical molecules with standard reference materials, cannot easily be transferred to much more complex high molecular weight proteins, such as used in this study. This particularly holds true for a very first PT performed in a technically diverse experimental surrounding. One of the most influencing factors is the choice of reference material, which is used to quantify concentration in the test samples. For BoNT no suitably qualified reference material is available and participating laboratories had to use BoNT from various sources and of different qualities and activities. As for many biological products, their specific activity and stability can be influenced considerably by their composition (e.g. holotoxin *versus* complex forms), impurities, buffer, as well as by the strain used, growth conditions and the specific purification process, all resulting in lot-to-lot variation of the final product. Such variability, even from the same supplier for different batches of toxins are not uncommon (note different lots of Metabiologics’s BoNTs used in the Experimental Section below) and have been observed by others [50]. Compared to commercially available purified toxins with significant lot-to-lot variations, the recombinant material used for the calculation of the nominal concentration in this PT underwent a much more thorough characterization including amino acid analysis [34]. Due to the complex nature of BoNT and the diversity in technologies and assays used, even the well characterized therapeutic preparations of BoNT are not interchangeable [51,52]. Nevertheless, when comparing the *z*-scores achieved by the three reporting laboratories with the *z*-scores of the other laboratories, the *z*-scores were found to be reasonably close to the nominal concentration (*z* = 0), and many lay satisfactorily between −3 and 3 (please refer to Worbs *et al.* [35] for more details). Figure 3 displays the distribution of *z*-scores between −10 and 10 for (A) decreasing concentrations of BoNT/A (1001, 100, 9.9, and 0.5 ng/mL) and (B) for the matrix compatibility test of approximately 10 ng/mL BoNT/A in buffer, cow’s milk, meat extract, and human serum for the three reporting laboratories.

**Table 3:** Overview of quantitative results.

Sample	Matrix	Analyte	*x*_a_ (ng/mL)	*x*_ELISA 4_ (ng/mL)	*z*_ELISA 4_	*x*_LFA 2_ (ng/mL)	*z*_LFA 2_	*x*_ELISA 2_ (ng/mL)	*z*_ELISA 2_	*x*_Endopep ELISA 2_ (ng/mL)	*z*_Endopep ELISA 2_
**S1**	meat extract	BoNT/A	10.5	20.0	3.6	26.0	5.8	15.3	1.8	13.9	1.3
**S2**	0.1% BSA/PBS	BoNT/A	9.9	18.8	3.5	29.6	7.8	12.9	1.2	15.1	2.1
**S3**	0.1% BSA/PBS	none	0.0	0.0	n/a	0.0	n/a	0.0	n/a	0.0	n/a
**S4**	0.1% BSA/PBS	BoNT/E	10.9	12.5	0.6	20.7	3.6	6.56	−1.5	21.3	3.8
**S5**	meat extract	BoNT/A	108.0	205	3.5	173	2.4	148	1.4	161	1.9
**S6**	0.1% BSA/PBS	BoNT/B	9.0	8.00	−0.4	9.30	0.1	7.91	−0.5	27.1	7.9
**S7**	0.1% BSA/PBS	BoNT/A	100.0	216	4.5	195	3.7	163	2.5	191	3.6
**S8**	0.1% BSA/PBS	BoNT/A	4.7	9.65	4.1	10.5	4.8	7.47	2.3	7.69	2.4
		BoNT/B	4.5	4.35	−0.1	4.50	0.0	4.10	−0.4	13.1	7.5
**S9**	0.1% BSA/PBS	BoNT/A	0.5	0.770	2.2	1.25	6.0	0.718	1.8	0.875	3.0
**S10**	milk	BoNT/A	10.3	17.1	2.6	16.0	2.1	16.3	2.3	13.8	1.3
**S11**	serum	BoNT/A	9.8	17.7	3.1	14.2	1.7	15.4	2.2	22.0	4.9
**S12**	0.1% BSA/PBS	BoNT/A	1001.0	1664	2.6	2173	4.6	1487	1.9	1542	2.1
**S13**	milk	BoNT/A	112.0	211	3.5	218	3.7	162	1.7	130	0.6

*x*_a_: assigned value; *x*: observed value; *z*: *z*-score, n/a: not applicable.

The immunoassays in this study achieved good results across a broad range of concentrations including the lowest concentration of 0.5 ng BoNT/A per mL (Figure 3A). It should be noted that the lowest concentration of 0.5 ng/mL could not be detected or quantified by a number of other participants excluding the three participants reporting here applying other immunoassays as well as other methods [35]. Nevertheless this concentration (0.5 ng/mL) cannot be considered as particularly low, as it is highly relevant for detection of toxin in patient serum [23,27,53]. Also, this concentration is readily detectable by other techniques such as the mouse bioassay, with a known detection limit of 5–10 pg/mL of BoNT/A or B [54].

The reported detection limits achieved by the ELISAs and LFAs used in this study are in all the pg/mL range. A range that can be expected by sandwich immunoassays employing colorized substrates as a read-out. There are a number of more sensitive and sophisticated read-outs such as enhanced chemiluminescence, electro-chemiluminescnce or immune-PCR to increase the sensitivity even further [55,56,57].

The applied immunoassays in this publication were compatible with different types of matrices (Figure 3B), while problems with matrix effects have been identified within this PT for other immunoassays and methods [35]. Of note is the human serum sample, present in S11, which resulted in the highest number of false results [35]. Matrix compounds can give rise to false positive or false negative results and have been observed by various techniques: not only standard immunoassays but also assays utilizing mass spectrometry, surface plasmon resonance or aptamer-based detection [21,58,59,60,61,62]. The majority of problems concerning specificity and matrix effects are dependent on the antibodies used. Polyclonal antibodies (pAbs), in addition to antibodies directed against the desired antigen, also usually contain antibodies directed against numerous unknown antigens, which can lead to false positive signals in the assays. The number and diversity of epitopes recognized by any pAb can be both an advantage and disadvantage. Whereas recognition of several epitopes can reduce failure to detect antigenic variants with slightly altered amino acid composition or with post-transcriptional modifications, binding to multiple epitopes can increase the possibility of recognizing unrelated proteins, containing an identical or similar epitope, and can lead to false positive results. Some of the drawbacks of pAbs can be overcome by use of monoclonal antibodies (mAbs), which recognize a single epitope. However, the strict antigenic specificity of mAbs can be a potential drawback when antigenic variants or subtypes have to be detected.

In recent years, more than 40 BoNT subtypes have been identified across the seven BoNT serotypes which differ between 1 and 36% at amino acid level [9,48]. This variability within the serotypes poses a challenge towards all kinds of genetic and immunological detection methods. Strictly speaking, all assays which rely on antibodies either for immunoenrichment or detection have to be tested for reactivity with all known subtypes or sequence variants, unless the exact antigenic epitope is known. However, such comprehensive analysis has been difficult to conduct due to the restricted access to known subtypes. There are only selected examples where antibodies have been tested against a number of subtypes [25,63,64,65,66]. For qualitative detection certain functional assays, like the mouse bioassay, hemidiaphragm or cell culture assays it can be assumed that sequence variation will not be of major impact as long as the BoNT-subtype/variant is effective on the underlying species (e.g., human, mouse, rat). Whereas on quantitative level the above mentioned functional assays can vary considerably depending on the assay and subtype used. In the frame of this PT the subtypes BoNT/A1, BoNT/B1, and BoNT/E1 have been used as recombinant material for the preparation of samples [34], strictly meaning that the results obtained are valid for these subtypes only and cannot necessarily be extrapolated to any other subtypes of a serotype. The issue how subtypes affect qualitative and quantitative results will have to be addressed in future exercises.

Furthermore, it has to be mentioned that in this PT BoNT holotoxins were tested. In nature the BoNTs are released in complexed form associated with the NTNHA as M-complex (also called M-PTC) or as a higher molecular weight L-complex (L-PTC) with additional hemagglutinins attached to the M-complex. Only recently the exact composition and structures of the M- and L-complexes have been elucidated [3,4,6]. The formation of the L-complex is restricted to sero- (B, C, D, and G) and BoNT/A subtypes (A1, A5), which occur in a hemagglutinin containing neurotoxin gene cluster (*ha^+^orfX^−^*). Serotypes E and F as well as the subtypes A1, A2, A3, A4, A6, and A8 of BoNT/A occur in a neurotoxin gene cluster where the three hemagglutinin genes are replaced by three *orfX* genes (*ha^−^orfX^+^* cluster), of yet not understood function [41,48]. It has been acknowledged that the association between BoNT and the complex proteins is pH sensitive. At low pH the complex is stable whereas above neutral pH the complex readily dissociates releasing free BoNT holotoxin [67,68,69]. Therefore the BoNT can be found as the M- or L-complex in many food matrices, the free BoNT holotoxin is most likely present in more basic foods and in serum. For diagnostic purposes therefore, detection of both the free BoNT holotoxin and in its complexed forms are equally important. This important point has to be addressed for any kind of test during assay development. The above-mentioned immunoassays have been qualified for the detection of BoNT holotoxin and the various BoNT complexes during assay establishment and validation.

The undisputed strength of immunoassays lies in their ease of use and high-throughput capability, which make them ideal screening tools. Numerous immunoassays for BoNT detection have been described, some of which have been validated for use with clinical, environmental or food matrices (reviewed in [11]), but very few were tested against a set of unknown samples in a ring trial or PT [70,71]. CEA and RKI used classic sandwich ELISA developed in-house for the effective quantitation of the different BoNT serotypes of unknown samples within this PT. A disadvantage of the classic plate-based sandwich ELISA is the lack of multiplexing capacity. This has been overcome by array-based approaches [72,73] or bead-based suspension assays [74,75], analogous to the one used by RKI for screening, which drastically reduces the sample volume consumed. Usually, sandwich ELISAs take between 3 to 8 h to return results, which created a demand for faster assays. Immunochromatographic tests, either column-based or as LFA, are simple and very fast immunoassays that have been developed for a wide range of analytes including different BoNTs [76,77,78,79,80,81,82,83,84,85]. Compared to sandwich ELISA they lack washing steps and thus might be more prone to matrix effects [58,76]. Moreover, their sensitivity can be inferior (ng/mL-range), however there are some exceptions with LODs in the low pg/mL range including the so far unpublished in-house LFA from CEA and others [83,84].

It has to be acknowledged that immunoassays deliver signal intensities, but not unambiguous confirmation on the presence of an antigen or biologically active antigen. False positive and false negative results can occur and cannot always be accounted for by proper controls such as the inclusion of an irrelevant antibody. If unequivocal detection is required, immunoassays must be accompanied by additional confirmatory methods such as mass spectrometry.

In addition to detection of the protein itself, the enzyme activity of the BoNT light chain can be assessed by measurement of its endopeptidase activity. Depending on the serotype, BoNTs cleave a specific position within one (or two for BoNT/C) of the SNARE proteins, SNAP-25, VAMP or syntaxin [9]. In endopeptidase assays the cleaved fragments of the SNARE proteins or of corresponding peptides are detected. This can be achieved directly by for example Western blotting, ELISA, MS-based methods, or indirectly by using fluorescence or otherwise labeled peptides (for review see [11,86]). Due to the enzymatic reaction, these assays can be very sensitive, sometimes surpassing even the mouse bioassay. NIBSC had developed such highly sensitive ELISA-based endopeptidase assays in the past [26,27,28,29,42,43] and successfully applied them for the detection, discrimination and quantitation of BoNT activity in this PT. Functional methods have the advantage that they relate to the function of the protein and as such refer to its integrity and activity rather than detecting its presence. It is worth noting that quantitative results from NIBSC indicated significant differences in detection of BoNT/A between the first and the second assay, performed up to 13 days apart. These results could potentially indicate loss of activity over time and the need for caution in use of non-functional assay methods in prediction of stability. However, no obvious drop in activity was evident from the data submitted based on other functional activity assays such as the mouse bioassay, hemidiaphragm or the Endopep-MS assay [35]. Within this PT, sample stability was confirmed by sandwich ELISA for samples stored over four weeks at 4 °C; this method has been selected on the basis of its high-throughput capability to analyze more than hundreds samples on a single day [35]. Future exercises have to consider this issue and the question if and how more than hundreds of samples can be tested for their functional stability on a single day. With activity assays it can be judged whether a protein is still active or even partially inactivated (for example by chemicals such as formaldehyde) which has major implications for hazard or risk assessment. Nevertheless, functional methods addressing only a portion of the whole biological activity of BoNT (receptor binding, internalization, translocation of the heavy chain or enzymatic activity of the light chain) do not detect all key activities essential for toxicity *in vivo* and can return false signals in the presence of *in vivo* inactive protein fragments such as the BoNT light chain. The limitation of the first generation of endopeptidase assays, as demonstrated in this PT study, is notable interference from the serum proteins on the substrate, observed particularly for BoNT/E assay. To overcome this limitation, the next generation of endopeptidase assays should combine a binding step, using an antibody directed against the BoNT heavy chain, followed by a substrate cleavage step and ultimate detection of the cleaved substrate by a neo-epitope-specific antibody, as previously reported for BoNT/A [28]. Dual approaches of an immuno-enrichment step including washing steps combined with an endopeptidase assay facilitate the detection of intact BoNT molecules and limit also matrix interferences thereby allowing detection from very crude matrices like feces [18,21]. Such an approach assessing enzymatic activity and presence of the receptor binding domain in one assay format has been successfully explored for detection of BoNT/A in a clinical sample [27] and could be applied to other BoNT serotypes, subject to identification of suitable monoclonal antibodies or receptor ligands.

There have been only a few other reports in which BoNT-immunoassays were assessed in ring trials or inter-laboratory exercises. The US Food and Drug Administration (FDA) and the CDC conducted in-depth validation and two collaborative studies, one with supernatants from cultured strains and the other with four spiked milk samples [71,87]. In both trials, individual sandwich ELISAs detecting BoNT/A, B, E, and F were used. Overall assay performance compared to the mouse bioassay was good with 94.7 and 92.3% clinical sensitivity (true positives/(true positives + false negatives)), respectively. However, some problems with matrix effects and cross-reactivity occurred, highlighting once again need for a case-by-case approach as a crucial factor in assay development and validation.

## 3. Experimental Section

### 3.1. CEA

#### 3.1.1. Ethics Statement

All experiments used for the generation of antibodies were performed in compliance with the French and European regulations on care and protection of Laboratory Animals (EC Directive 86/609, French Law 2001-486, 6 June 2001) with agreement n°91–416 delivered to Dr. S. Simon by the French Veterinary Services and CEA agreement D-91-272-106 from the Veterinary Inspection Department of Essonne (France).

#### 3.1.2. Reagents

Sandwich ELISA were performed on Nunc Maxisorp 96-well microtiter plates (VWR, Fontenay-sous-Bois, France) and all reagents were diluted in Enzyme ImmunoAssay (EIA) buffer (0.1 M potassium phosphate buffer pH 7.4 containing 0.15 M NaCl, 0.1% (*w*/*v*) bovine serum albumin (BSA) and 0.001% (*w*/*v*) NaN_3_). Plates coated with proteins were saturated in ELISA buffer (18 h at 4 °C) and washed with washing buffer (0.01 M K phosphate buffer pH 7.4 containing 0.05% (*v*/*v*) Tween 20).

#### 3.1.3. Description of the Performed Tests 

Monoclonal murine antibodies raised against BoNT/A have been obtained as described previously [69], using the recombinant binding domain (*C*-terminus part of heavy chain) of BoNT/A as immunogen. Evaluation of antibody sensitivity and specificity either in sandwich ELISA or lateral flow immunoassays (LFA) allowed the selection of the best mAbs pairs for each type of test (TA18/TA13* for sandwich BoNT/A ELISA and TA12/TA9* for BoNT/A LFA, TB6/TB17* for both BoNT/B tests, and TE81/TE49* for both BoNT/E tests, where * indicates the conjugate antibody).

#### 3.1.4. Sandwich ELISA

Immunometric assays were performed in 96-well microtiter plates coated with one of the mAbs as previously described [69]. The assays were performed using a “simultaneous procedure”, involving addition of 100 μL of samples or standard solutions of BoNT/A, B or E (all from Metabiologics) diluted in assay buffer (0.1 M Tris-HCl pH 8, 0.15 M NaCl, 0.1% (*w*/*v*) BSA, 0.1% (*w*/*v*) NaN_3_) to each well together with 100 μL of mAb-AChE conjugate (the labeling was performed as described in [88]) at a concentration of 5 EU/mL for an 18-h incubation at 4 °C. Each standard preparation was prepared in duplicate and diluted 8-fold in the blank (buffer alone) in order to determine the minimum detectable concentration. After incubating, the plates were extensively washed and solid-phase bound AChE activity was revealed by addition of 200 μL of Ellman’s reagent for a 30-min to 3-h reaction [89].

#### 3.1.5. Lateral Flow Immunoassays

The colloidal-gold-labeled mAb and the strips were prepared as previously described [90]. During experiment, 100 μL of samples diluted in assay buffer (0.1 M Tris-HCl pH 8, 0.15 M NaCl, 0.5% (*v*/*v*) Tween 20, 1% (*w*/*v*) 3-[(3-Cholamidopropyl)dimethylammonio]-1-propanesulfonate [CHAPS], 0.1% (*w*/*v*) NaN_3_) were mixed with 10 μL of colloidal-gold-labeled antibodies (20 μg/mL) in the wells of a 96-well microtiter plate. After 10 min incubation of the mixture at RT, the strips were inserted into the wells. The capillary migration from the bottom of the sample pad to the absorption pad in the upper position lasted for about 30 min. The signal intensities of the test and control lines were visually estimated or read by an ESE Quant reader (Qiagen, Courtabœuf, France) parametrized with a standard curve.

### 3.2. RKI

#### 3.2.1. Ethics Statement

Antibodies were generated in laboratory mice, rabbits and hens. All experiments were performed in compliance with the German Animal Welfare Act (*Tierschutzgesetz*) and European legislation for the protection of animals used for scientific purposes (Directive 2010/63/EU). All animal experiments were authorized under license H109/03 and overseen by the local state authorities (*Landesamt für Soziales und Gesundheit*) and the institutional Animal Welfare Officer.

#### 3.2.2. Antibodies

Apart from previously published antibodies [36,37,40], mAb and rabbit and chicken pAb were raised against BoNT/E or BoNT/F toxoids made from purified toxins (Metabiologics) or *C*-terminal BoNT heavy chain fragments (Toxogen, Hannover, Germany) as previously described [36,37]. All antibodies were tested for cross-reactivity against purified BoNT holotoxins and BoNT complexes (all from Metabiologics, Madison, WI, USA) and other proteins including ricin [91], abrin and SEB (both from Toxin Technologies, Sarasota, FL, USA) and bovine serum albumin (BSA). Detection antibodies were biotinylated using Biotinamidohexanoyl-6-aminohexanoic acid *N*-hydroxysuccinimide (Biotin-X-X-NHS) ester (Sigma-Aldrich, Munich, Germany) dissolved to 13.4 mM in DMSO. To 500 μL of antibody (1 mg/mL in PBS), 50 μL freshly prepared 1 M NaHCO_3_ were added and mixed. For a molar ratio of ten (antibody:biotin) 2.5 μL 13.4 mM Biotin-X-X-NHS were added and incubated end-over-end for 1 h. Coupling reaction was stopped by the addition of 10 μL 10% (*w*/*v*) NaN_3_. Biotinylated antibody was separated from unbound Biotin-X-X-NHS and salts by size exclusion chromatography over a PD10 column (GE Healthcare, Uppsala, Sweden) with PBS containing 0.05% (*w*/*v*) NaN_3_.

#### 3.2.3. Multiplex Bead-Based ELISA

Antibodies were covalently coupled to fluorescent magnetic beads (xMAP MagPlex Microspheres, Luminex Corp., Austin, TX, USA) according to the manufacturer’s instruction. For the 6-plex bead assay, antibodies against ricin (mAb R109), SEB (mAb S419), BoNT/A (mAb A1688; directed against heavy chain), BoNT/B (mAb B279; directed against light chain), BoNT/E (rabbit pAb KE97), and BoNT/F (mAb F220; directed against light chain) were coupled to different xMAP regions [37]. As standard purified BoNTs, ricin and SEB were used in serial half-log dilutions from 1 μg/mL down to 3.16 pg/mL. PT samples were either used undiluted or in consecutive 10-fold dilution series from 1:10 to 1:10,000 in assay buffer. Assay was carried out as previously described [37] with 1000 beads per 50 μL sample volume. For detection, biotinylated antibodies against ricin (mAb R18), SEB (mAb S1001), BoNT/A, B, E (horse trivalent antitoxin Behring, Novartis, Marburg, Germany), BoNT/E (mAb E136; directed against heavy chain), and BoNT/F (mAb F754; directed against light chain) were used. Measurement was carried out on a Bio-Plex 200 instrument and Bio-Plex Manager Software (Bio-Rad, Munich, Germany) and concentration calculated from the standard curve.

#### 3.2.4. Singleplex Sandwich ELISA

ELISAs were performed as previously reported [37,39]. In short, Nunc Maxisorp plates (Thermo Scientific, Brunswick, Germany) were coated over night with 10 μg/mL of capture antibody against BoNT/A (A1688), BoNT/B (B279) or BoNT/E (KE97). Plates were washed and non-specific binding blocked with casein blocking buffer (Senova, Weimar, Germany) for 1 h. Duplicates of two-fold serial dilutions of PT samples in PBS containing 0.1% (*w*/*v*) BSA were incubated for 2 h. Purified BoNT holotoxins (Metabiologics) were used as standard in duplicate. Their stated activity was 2.7 × 10^8^ LD_50_/mg for BoNT/A, 1.2 × 10^8^ LD_50_/mg for BoNT/B, and 3.0 × 10^5^ LD_50_/mg for BoNT/E. After washing, bound antigen was detected by a 1-h incubation with biotinylated antibody against BoNT/A (mAb HcA78, directed against heavy chain), BoNT/B (horse trivalent antitoxin Behring) or BoNT/E (mAb E136). Excess antibody was washed away followed by an incubation for 1 h with streptavidin-polyHRP conjugate (Senova). After final washing, signal was generated by adding 100 μL 3,3ʹ,5,5ʹ-Tetramethylbenzidine (TMB) solution (SeramunBlau slow; Seramun, Heidesee, Germany) and reaction stopped by the addition of 100 μL 0.25 M H_2_SO_4_. Absorbance was measured at 450 and 620 nm. Differences in absorbance at 450 and 620 nm were plotted against the log concentration of the BoNT standard and fitted against a sigmoidal dose response curve (4-parametric non-linear regression analysis) in Prism 5.04 (GraphPad, La Jolla, CA, USA). LOD was calculated from the regression curve as the mean of blank signals plus 3.29-times standard deviation (SD) of blank signals (99% confidence interval). LLOQ and ULOQ flank the “linear range” of the sigmoidal curve between the inflection points of the 1st derivative of the sigmoidal regression curve and were computed as the maxima and minima of the 2nd derivative.

### 3.3. NIBSC

#### 3.3.1. Ethics Statement

Antibodies were generated in rabbits and method of antibody preparation complied with UK Home Office project license (PPL # 80/2634) for Research on Bacterial Products used in Medicine. All experiments on animals at NIBSC (UK) were approved by the local animal research oversight committee, the Animal Welfare and Ethics Review Body.

#### 3.3.2. Reference Toxins

Purified hemagglutinin free botulinum type A1, B1 and E3 toxins were purchased from Metabiologics (Madison, WI, USA) with stated activity of 2.3 × 10^8^ LD_50_/mg (Hall strain), 2 × 10^8^ LD_50_/mg (Okra strain), and 3 × 10^5^ LD_50_/mg (Alaska strain), respectively. Only BoNT/E3 was trypsinzed and activity determined as 6 × 10^7^ LD_50_/mg. Toxins were diluted to 20,000 LD_50_/mL (87 ng/mL) for BoNT/A; 12,560 LD_50_/mL (306 ng/mL) for BoNT/B and 20,000 LD_50_/mL (100 ng/mL) for BoNT/E in 50 mM gelatin (0.2% *w*/*v*) phosphate buffer pH 6.5 and stored frozen at −80°C until use.

#### 3.3.3. Endopeptidase ELISA

The endopeptidase immunoassays for BoNT/A and BoNT/E were performed as previously reported [43] and suitably modified for BoNT/B. Briefly, all test samples and reference toxins were diluted in appropriate assay reaction buffer (50 mM HEPES with 20 μM ZnCl_2_, pH 7.0) containing 0.5% (*v*/*v)* Tween 20 with either 5 mM (*2S,3S*)-1,4-bis(sulfanyl)butane-2,3-diol (DTT) (for BoNT/A and /B) or 2.5 mM DTT (for BoNT/E). Doubling dilutions were performed across the 96 well polystyrene ELISA plates (Nunc Maxisorp) previously coated with 50 μL per well of either 3 μg/mL of SNAP-25 (amino acids 137–206) or 10 μg/mL of VAMP2 (amino acids 60–94) peptide substrate for BoNT/A/E or B assays, respectively. The plates were then incubated for 18 h either at room temperature (for BoNT/A) or at 37 °C (for BoNT/B and E) and washed three times with PBS supplemented with 0.05% (*v*/*v*) Tween 20.

Cleavage by the toxin was detected by addition of 100 μL per well of purified rabbit antibody recognizing only newly exposed epitopes generated by each of the toxin serotypes (*i.e.*, SNAP25_190–197_, SNAP25_173–180_ or VAMP2_77–84_). Antibodies were diluted in 2.5% (*w*/*v*) skimmed milk powder in PBS (MPBS) and plates incubated for up to 90 min at room temperature. The plates were then washed three times in PBST and 100 μL/well of goat anti-rabbit conjugate (for BoNT/A and /E: Sigma Aldrich (Poole, UK), coded A0545 at 1 in 2000; for BoNT/B: Amersham Pharmacia Biotech RPN4301 batch 5 AMD-HRP at 1 in 20,000) was added. After a final washing, ABTS substrate solution (2,2′-azino-bis(3-ethylbenzothiazoline-6-sulfonic acid) diammonium salt, (Sigma Aldrich, Poole, UK), 0.05% (*v*/*v*) of 30% (*w*/*v*) hydrogen peroxide solution, 50 mM citric acid pH 4.0) was added at 100 μL per well. The color reaction was evaluated by determining absorbance at 405 nm with a plate reader (Multiskan MS, Labsystems, Helsinki, Finland). The estimated toxin concentration was calculated using Combistats parallel-line analysis software (European Directorate for the Quality of Medicines and HealthCare, Strasbourg, France).

## 4. Conclusions

Immunoassays cannot unambiguously detect the presence of an analyte. However, they offer a number of advantages such as versatility, rapid turnaround time, ease of use, flexibility, high throughput and high sensitivity. Their success is strongly dependent on the quality of antibodies used as well as on the scope of the validation scheme applied.

Here we describe different immunoassay approaches applied for the detection, serotyping and quantification of three BoNT serotypes (BoNT/A, B, and E) from 13 blinded samples containing different toxin concentrations and supplied in different matrices within an international PT. Since the actual BoNT concentration of a sample is an important factor for risk assessment, PT participants were asked to deliver not only qualitative results including serotyping but also quantitative results for each sample. All described immunoassays were able to correctly identify the serotype present in each sample with a single exception, highlighting a reasonable standard in immunoassay detection quality. Sensitivity and robustness of the described approaches against matrix effects were highlighted on the basis of decreasing analyte concentrations and varying sample matrices. Based on the different assay principles, this publication discusses advantages and limitations of the selected immunoassays and offers guidance to the reader for the selection, development and characterization of their own approaches. Relevant parameters (e.g. antibodies, cross-reactivity, or matrix effects) were discussed. This study indicated that precise quantification was more challenging compared to qualitative reporting. Whilst the quantitative results were in the right order of magnitude, they had the tendency towards a positive deviation from the nominal values. One of the most influencing factors for quantification is the use of different reference materials. The three laboratories in this study obtained reference material from the same commercial source (Metabiologics Inc. Madison, WI, USA) but used different lots of different purity and specific activity for quantification. One important factor for future harmonization and standardization of analytical approaches will be the accessibility of a precisely characterized (certified) reference material of known protein content and biological activity. It can be expected that such reference materials will improve quantification and allow for a more robust comparison of analytical approaches.
